# Stress echocardiography in heart failure patients: additive value and caveats

**DOI:** 10.1007/s10741-024-10423-9

**Published:** 2024-07-26

**Authors:** Maria Concetta Pastore, Alessandro Campora, Giulia Elena Mandoli, Matteo Lisi, Giovanni Benfari, Federica Ilardi, Alessandro Malagoli, Simona Sperlongano, Michael Y. Henein, Matteo Cameli, Antonello D’Andrea, on behalf of the Echocardiography Working Group of the Italian Society of Cardiology (SIC)

**Affiliations:** 1 Department of Medical Biotechnologies, Division of Cardiology, University of Siena, Viale Bracci1 , Siena, Italy; 2 Department of Cardiovascular Disease – AUSL Romagna, Division of Cardiology, Ospedale S. Maria Delle Croci, Viale Randi 5, 48121 Ravenna, Italy; 3 Section of Cardiology, Department of Medicine, University of Verona, Verona, Italy; 4 Department of Advanced Biomedical Sciences, Division of Cardiology, Federico II University Hospital, Via S. Pansini 5, 80131 Naples, Italy; 5 Division of Cardiology, Nephro-Cardiovascular Department, Baggiovara Hospital, Modena, Italy; 6 Division of Cardiology, Department of Translational Medical Sciences, University of Campania Luigi Vanvitelli, Naples, Italy; 7 Department of Public Health and Clinical Medicine, Umeå University, Umeå, Sweden; 8 Department of Cardiology, Umberto I Hospital, 84014 Nocera Inferiore, SA Italy

**Keywords:** Stress echocardiography, Heart failure, Valvular heart disease, Exercise, Diagnosis

## Abstract

Heart failure (HF) is a clinical syndrome characterized by well-defined signs and symptoms due to structural and/or myocardial functional impairment, resulting in raised intracardiac pressures and/or inadequate cardiac stroke volume at rest or during exercise. This could derive from direct ischemic myocardial injury or other chronic pathological conditions, including valvular heart disease (VHD) and primary myocardial disease. Early identification of HF etiology is essential for accurate diagnosis and initiation of early and appropriate treatment. Thus, the presence of accurate means for early diagnosis of HF symptoms or subclinical phases is fundamental, among which echocardiography being the first line diagnostic investigation. Echocardiography could be performed at rest, to identify overt structural and functional abnormalities or during physical or pharmacological stress, in order to elicit subclinical myocardial function impairment e.g. wall motion abnormalities and raised ventricular filling pressures. Beyond diagnosis of ischemic heart disease, stress echocardiography (SE) has recently shown its unique value for the evaluation of diastolic heart failure, VHD, non-ischemic cardiomyopathies and pulmonary hypertension, with recommendations from international societies in several clinical settings. All these features make SE an important additional tool, not only for diagnostic assessment, but also for prognostic stratification and therapeutic management of patients with HF. In this review, the unique value of SE in the evaluation of HF patients will be described, with the objective to provide an overview of the validated methods for each setting, particularly for HF management.

**Supplementary Information:**

The online version contains supplementary material available at 10.1007/s10741-024-10423-9.

## Introduction

Heart failure (HF) is the most common cause of death among cardiovascular (CV) diseases [1, 2] and its incidence continues to increase, mainly due to aging of the population. Therefore, new investigations have been developed and others are currently under evaluation to help improving prognosis and quality of life of HF patients. Stress echocardiography (SE) is a validated tool not only for the assessment of ischemic HF [3], but also for non-ischemic HF, such as or valvular heart disease [4] or unmasking significant diastolic dysfunction, allowing the identification of early stages of HF with preserved ejection fraction (HFpEF) [5]. This leads to early start of treatment, with considerable improvement of symptoms and quality of life. SE has also been used in advanced HF to optimize the selection of marginal donors with the aim of extending the pool of donors for heart transplantation [6].

The aim of the present review is to highlight the unique value of SE for the evaluation of HF patients, analyzing the advantages and disadvantages of the various SE modalities and describing different clinical applications in HF setting.

## Heart failure: pathophysiology of alterations induced by stress

### Valvular heart disease

Resting transthoracic echocardiography is the most used investigation for non-invasive evaluation of valvular heart disease (VHD). However, VHD dynamic component is significantly influenced by loading conditions, ventriculo-arterial coupling and subclinical myocardial dysfunction, which may be underestimated at rest. Thus, SE may add diagnostic information in VHD, through the evaluation of clinical response and dynamic changes in ventricular and valvular function during exercise/stress, e.g. in patients reporting HF exertional symptoms but not showing significant VHD at resting echocardiography.

In mitral valve disease, the increase in heart rate (HR) causes a fall in filling time and LV preload and an increase in left atrial and pulmonary arterial pressures. This may explain symptoms like shortness of breath on exertion, disproportionate to the resting degree of valvular disease, which may in severe cases lead to HF and unexplained acute pulmonary edema [7, 8].

Chronic aortic regurgitation (AR) results in increased LV pressure and volume overload and chronically HF. Symptoms induced by AR have an insidious and late onset. The onset of symptoms at rest is associated with bad prognosis and a reported annual mortality as high as 10–20%, thus Therefore, early identification of AR symptoms by SE may be crucial for its management [9].

Moreover, the use of SE is recommended as part of the diagnostic algorithm of aortic stenosis (AS), particularly in low-flow-low-gradient disease [7]. The presence of AS causes a chronic pressure overload, resulting in the development of concentric LV hypertrophy to maintain an adequate stroke volume (SV) and diastolic dysfunction. Exercise hemodynamics in patients with moderate-severe AS are usually abnormal even in patients without symptoms. Although in these patients resting CO could be normal, its increase with exercise is limited and is primarily mediated by an increase in HR. This results in a short systolic ejection interval with little change in SV and an increase in the velocity of the aortic jet and transvalvular gradient. Initially, aortic valve area tends to slightly increase with exercise (0.2 cm^2^ on average), underestimating symptoms. With worsening AS, aortic valve area does not significantly increase with exertion, while a further increase in jet velocity and transvalvular gradient could be demonstrated by SE (“true severe AS”) [8, 10]. Furthermore, patients with AS also develop an abnormal blood pressure (BP) response to exercise (increase by < 10 mmHg), which indicates severe valve obstruction, and, typically, this corresponds to the onset of symptoms [10].

### Diastolic HF

The use of SE in diastolic HF is based on the so-called “cardiac reserve”, defined as cardiac response to increased preload enhancing CO without significant increase in LV filling pressure, resulting from contractile reserve and relaxation (diastolic) reserve. The impairment of relaxation properties is called “diastolic dysfunction (DD)” and, if leading to HF symptoms is regarded as “diastolic HF” or “HFpEF”, which has gained increasing importance in recent years. In fact, it is the main cause of hospitalization in 40% of patients presenting with symptoms of HF [11, 12].

Patients with HFpEF or DD and little or no sign of congestion at rest may present with exercise intolerance for several reasons: 1) high LV diastolic/pulmonary venous pressure during stress causing reduction in lung compliance, increase breathing work and dyspnea [13]; 2) many patients present with LV concentric hypertrophy with small end-diastolic LV volume, so during exercise the Frank-Starling mechanism limits these ventricles, with compromised SV and CO [14, 15]; 3) subtle and latent contractile abnormalities are typically present in many of these patients, in whom, however, DD is the dominant feature [13]. The investigation of DD by diastolic SE is suggested in the latest HF guidelines of the European Society of Cardiology (ESC) [16] in the presence of equivocal resting echocardiographic and laboratory markers, as an attempt to establish the diagnosis of HFpEF.

### Ischemic HF

One of the most conventional applications of SE is to find myocardial ischemia as a transient, regional imbalance between augmented myocardial oxygen demand and inadequate supply. Myocardial ischemia results in a typical cascade of events with well-defined time sequence: regional mechanical dysfunction (with a reduction of segmental motion and thickening) can be early detected by SE, ECG changes and the onset of chest pain occurs later [17]. SE may also be used for follow-up evaluation of patients with CAD with residual coronary stenosis or new onset of symptoms [16]. In the absence of coronary artery disease (CAD), coronary flow reserve (CFR) can be reduced because of microvascular disease, causing angina with ST-segment depression and regional wall motion thickening (RWMT) abnormalities during stress [18, 19].

The loss of myocytes secondary to myocardial necrosis in CAD is the leading cause of HF. Therefore, noninvasive identification of the extent of myocyte loss and estimation of the extent of segmental viability is particularly useful for clinical decision making in patients with ischemic LV dysfunction.

## Stress echo – methodology

### Protocols and stressors

SE provides dynamic evaluation of myocardium under conditions of physiological (exercise) or pharmacological (inotrope, vasodilator) stress to unveil structural/functional abnormalities, absent at rest, such as wall motion abnormalities, VHD, or other hemodynamic disturbances [20–23].

**Fig. 1:**
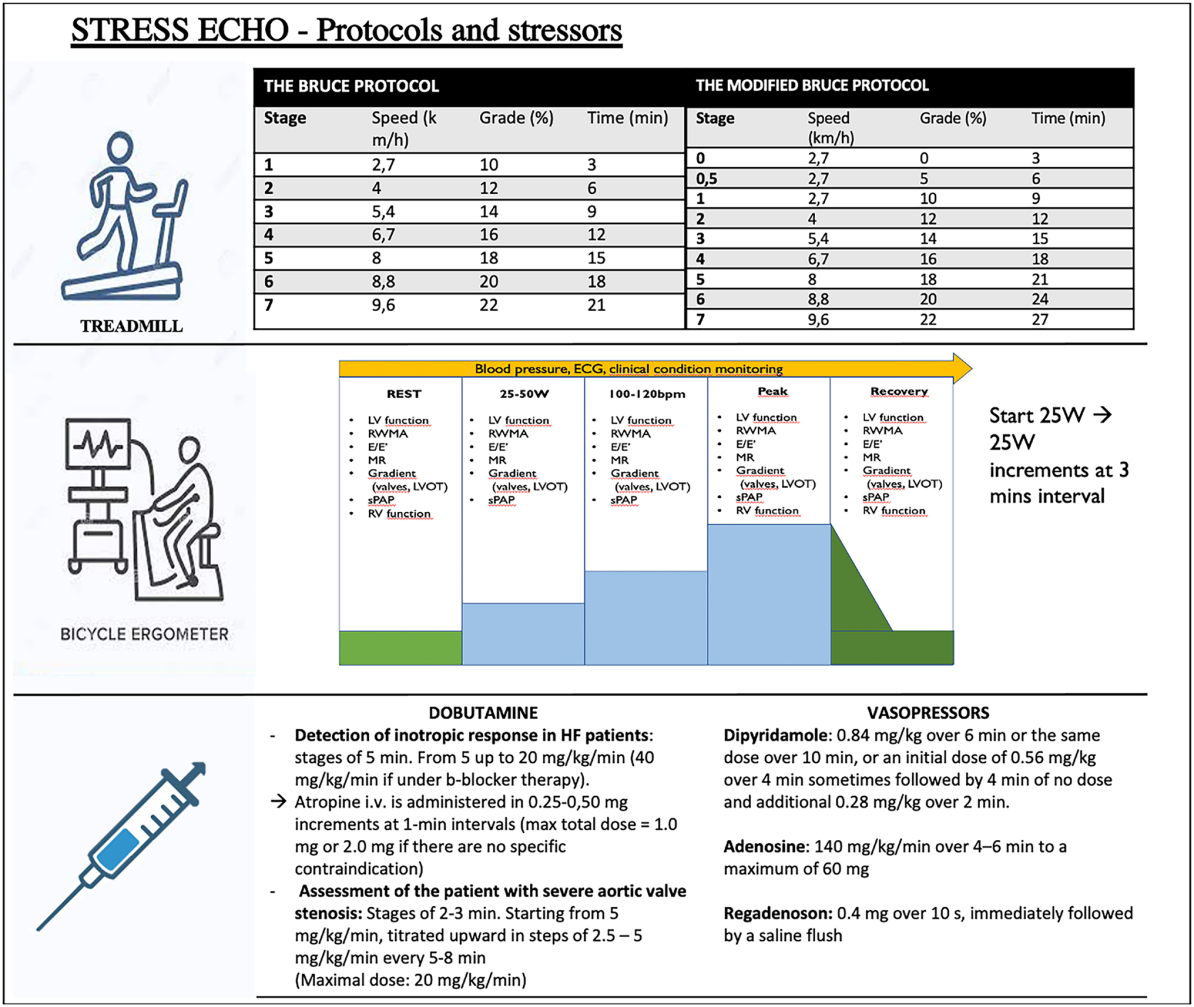
Stress echo – protocols and stressors description of the Bruce and the modified Bruce protocol, presenting characteristics of all their stages [25]; description of bicycle ergometer SE protocol, with the main echocardiographic parameters to be acquired at each stage; main pharmacological stressors used in stress echocardiography with their administration protocols. HF, heart failure; LV, left ventricle; LVOT, left ventricular outflow tract; MR, mitral regurgitation; RV, right ventricle; RWMA, relative wall motion abnormalities; SPAP, systolic pulmonary artery pressure

Physical exercise SE (treadmill or bicycle) should be the preferred method since it reflects physiological hemodynamic response to exercise and provides information on exercise capacity [24]. For the treadmill test, Bruce/modified Bruce protocols or bicycle ergometer SE are used [25] (Fig. 1).

In patients unable to undertake physical exercise or those with abnormal resting RWMT, pharmacological SE should be chosen [24]. The commonly used stressors are dobutamine and vasodilators. In Fig. 1, information about the administration protocols of pharmacological stressors during SE are reported. Dobutamine acts mainly on myocardial β-1 adrenergic receptors, increasing HR and contractile function, with a consequent higher myocardial oxygen consumption. Myocardial contractility increases over four-fold in healthy subjects and much less (less than two-fold) in patients with HF [26]. The dobutamine activation of β-2 adrenergic receptors, through a vasodilatory effect, may contribute to a mild decrease in BP at higher doses. Compared with exercise SE, the increase in wall stress is lower, due to reduced recruitment of venous blood volume with dobutamine. During or soon after the maximum dose of dobutamine, atropine can be administered to increase heart rate and improved test-accuracy [24].

Vasodilator SE can be performed with dipyridamole, adenosine, or regadenoson. All these stressors share the same metabolic pathway. Dipyridamole increases endogenous adenosine levels, adenosine increases exogenous adenosine levels, while regadenoson acts directly on vascular A2A adenosine receptors, with higher receptor specificity, less test duration and less potential for complications. The activation of this pathway produces a small decrease in BP, modest tachycardia, and a mild increase in myocardial function [27, 28]. In the presence of a critical epicardial stenosis or microcirculation dysfunction, vasodilator administration causes a heterogeneity of coronary blood flow between stenotic and normal coronary arteries subtended areas. This provokes a supply–demand mismatch, and therefore, via steal phenomenon, a reduction in subendocardial flow in areas of coronary artery stenosis.

### Indications—when and which SE modality should be preferred

Patients able to perform physical exercise should undergo physical stress, as described in Table 1. This preserves the integrity of the electro-mechanical response and allows the correlation of symptoms with CV workload, wall motion abnormalities, and hemodynamic responses, such as pulmonary pressure and transvalvular flows and gradients. Semi-supine bicycle exercise is technically easier than upright bicycle or treadmill exercise, especially when multiple stress parameters are assessed at peak exercise.

**Table 1:** Stress echocardiography: When and which type should be preferred. Description of the main stressors suggested for different clinical indication

**INDICATION**	**QUERY**	**TYPE OF STRESS**
**DIASTOLIC FUNCTION**	Diastolic dysfunction ± SPAP increase as reason for HF symptoms and signs	**EXERCISE**
**HYPERTROPHIC CARDIOMYOPATHY**	LVOTO/diastolic dysfunction/ dynamic MR/inducible ischaemia	**EXERCISE**
**DILATED CARDIOMYOPATHY**	Contractile reserve, inducible ischaemia, diastolic reserve, SPAP change, dynamic MR, pulmonary congestion	**EXERCISE**
	Inotropic reserve, inducible ischaemia	**DOBUTAMINE**
**CARDIAC RESYNCHRONIZATION THERAPY**	Inotropic reserve, viability in paced area	**DOBUTAMINE**
**AORTIC STENOSIS**	Severe AS with no symptoms	**EXERCISE**
	Non-severe AS with symptoms	**EXERCISE; DOBUTAMINE**
	Low–flow, low-gradient AS	**DOBUTAMINE; EXERCISE**
**PRIMARY MITRAL REGURGITATION**	Severe MR with no symptoms	**EXERCISE**
	Non-severe MR with symptoms	**EXERCISE**
**SECONDARY MITRAL REGURGITATION**	Change in MR severity with exertion ± SPAP increase	**EXERCISE**
	Severe AR with no symptom	**EXERCISE**
**MITRAL STENOSIS**	Non-severe MS with symptoms	**EXERCISE**
**AORTIC REGURGITATION**	Non-severe AR with symptoms	**EXERCISE**
	Severe MS with no symptoms	**EXERCISE**
**MULTIVALVULAR DISEASE**	Discordance in between symptoms and severity of valve disease	**EXERCISE**
**AORTIC VALVE PROSTHESIS**	Stenosis/PPM with or without low flow	**EXERCISE; DOBUTAMINE**
**MITRAL VALVE PROSTHESIS**	Stenosis	**EXERCISE; DOBUTAMINE**
**MITRAL VALVE ANNULOPLASTY**	Iatrogenic MS	**EXERCISE; DOBUTAMINE**
**PULMONARY HYPERTENSION**	Symptoms and SPAP on exertion	**EXERCISE**

*AR* aortic regurgitation, *AS* aortic stenosis, *HF* heart failure, *LVOTO* left ventricular outflow tract obstruction, *MR* mitral regurgitation, *MS* mitral stenosis, *PPM* patient-prosthesis mismatch, *SPAP* systolic pulmonary artery pressure

Pharmacological stress is usually not able to replicate the complex exercise-triggered hemodynamic and neurohormonal changes [29–31]. Among all pharmacological stressors, dobutamine is the preferred for the evaluation of contractile function and flow reserve. Different protocols have been used to evaluate contractile reserve, changes in LV volumes and ejection fraction (EF), including low-dose (10 mg/kg/min) to high-dose (40 mg/kg/min) dobutamine SE. High-dose is the preferred method in ischemic HF, while in patients with non-ischemic HF there is no consensus on the optimal dobutamine protocol [32, 33].

Vasodilator SE may be preferred for combined assessment of wall motion and CFR, which may be impaired in dilated non-ischemic cardiomyopathy and hypertrophic cardiomyopathy [27, 28]. Dipyridamole SE is not often used to study contractile reserve, but may be useful if patients are under treatment with β-blockers. It is associated with less arrhythmias [33]. Regadenoson, due to its high cost, is usually preferred in patients with chronic obstructive pulmonary disease and asthma [24].

Standard pharmacological ischemia protocol consists of three stages (rest, intermediate, peak), while the presence of RWMT abnormality necessitates a four-stage viability protocol for assessment of biphasic response. In patients with permanent pacemaker, exercise SE or combination of pacing with pharmacological stress can be performed solely with appropriate programming of the permanent pacemaker (i.e. high-rate pacing [34, 35].

### Advantages/limitations

SE is a validated, non-invasive, available, bed-side, low-cost investigation and is widely used for its ability to assess segmental LV function continuously and simply on a beat-by-beat basis. Echocardiography performed with vasodilator or inotropic stress has proved to be a clinically useful test for the assessment of myocardial ischemia and viability in the evaluation of VHD and cardiomyopathies. Compared with the competing nuclear or cardiac magnetic resonance (CMR) techniques, SE offers comprehensive information on VHD, despite some well-known limitations. These include subjectivity, particularly for the interpretation of wall motion and thickening, with a significant inter- and intra-observer variability. The variability appears to be very small when baseline image quality is good but turns out to be significant for patients with poor acoustic window [36, 37]. There is also a significant degree of variability of interpretation of SE depending on the level of expertise of the operator and center experience [38].

## Stress echo – clinical applications in heart failure

In the latest guidelines of the European Society of Cardiology (ESC) for the diagnosis and treatment of acute and chronic HF, [16] SE role is mentioned for the evaluation of the underlying disease causing the HF, prognostic stratification and therapeutic management. The clinical applications of SE in HF setting could be categorized in two main subsets: SE in ischemic HF and SE in non-ischemic HF.

### Ischemic HF

SE can detect both myocardial ischemia and viability in patients with CAD [39]. Wall motion may visually and semi-quantitatively be graded in 16 segments on a 4 grade Likert scale: 1 = normal, 2 = hypokinesia, 3 = akinesia, and 4 = dyskinesia. Then, the sum of the scores of each segment constitutes wall motion score index. The preferred SE method for the detection of both ischemia and viability is high-dose dobutamine [40] (which may cause arrhythmias), while low- dose dobutamine is sufficient if the evaluation of ischemia is not required [41]. Ischemia is diagnosed if at least two segments in the same coronary territory degrade by at least one-point with high-dose dobutamine. Viability is detected when at least two dysfunctional segments in the same coronary territory improve at low-dose dobutamine by at least one-point or present a biphasic response at high-dose dobutamine infusion [42].

In the latest ESC HF guidelines exercise or pharmacological SE was suggested for the assessment of inducible ischemia [16]. Particularly, the guidelines state that non-invasive stress imaging [CMR, stress echocardiography or nuclear imaging may be considered (class IIb recommendation)] for the assessment of myocardial ischemia and viability in patients with CAD suitable for coronary revascularization [43], and these may serve as a guide for invasive coronary angiography, which may be considered (IIb) in patients with HF while reduced EF (HFrEF) with an intermediate to high pre-test probability of CAD in presence of ischemia on non-invasive stress test [44].

In fact, SE application for diagnosis of significant CAD has a sensitivity and specificity of 85% and 77% respectively. Besides, with a negative exercise SE, patients have a very low mortality and significant events rate (0.6–0.8% per patient/year), even with an intermediate-high pretest probability [45]. The outcome of patients with an abnormal SE compared to normal SE is significantly worse (survival 71.2% vs 92%, respectively) [3].

The identification of dysfunctional but viable myocardium with SE, with its subsequent revascularization, may allow improvement of regional and global cardiac systolic function [46–48]. Moreover, several non-randomized studies have evaluated the endpoint of death, suggesting that viability-guided revascularization might improve patients’ survival [49–51]. However, these results have not been confirmed in different prospective randomized trials [48, 52–56]. For this reason, current guidelines do not advocate (Class IIb indication) routine testing of myocardial viability to select revascularization of patients with HF. Considerable debate remains on how these results should be interpreted, [57] and viability assessment remains widely used in clinical practice.

Currently, the study of myocardial viability is suggested in the following clinical settings [58]:

- to guide revascularization of patients with HF, known CAD and wall motion abnormalities (ESC Class IIb Level B);
- to guide revascularization of patients presenting with HF and late presentation of acute coronary syndrome;
- to select optimal revascularization (percutaneous vs. surgical) strategy in patients with complex multivessel CAD;
- to decide upon percutaneous revascularization or medical therapy in patients with chronic total occlusions.

Furthermore, the study of myocardial viability is applicable even in VHD:

- to determine the mechanism of ischemic MR and guide decision making for valve replacement and revascularization in patients with ischemic MR, LVEF < 30% and an option for surgical revascularization (ESC Class IIa Level C);
- to determine the contractile response (low-dose dobutamine echocardiography) in low-flow AS (ESC Class IIa Level C).

In patients with normal LV kinetics at peak SE measuring CFR could provide an additional value. It is assessed as the ratio between stress to baseline peak diastolic coronary Doppler flow velocities, usually obtained from left anterior descending artery (LAD) images. In all types of SE, the main challenges are the visualization of LAD in the 4-chamber view and the acquisition of a correct Doppler signal, as the Doppler cursor has to be aligned with a moving small vessel. For these reasons, the most feasible modality of SE for CFR assessment proved to be dipyridamole. CFR has shown to be of additional prognostic value to RWMT assessment [10] and might be useful in microvascular disease recognition in different subsets of patients, such as diabetics, hypertensives, and those with hypertrophic or dilated cardiomyopathy [3].

Importantly, an emerging diagnostic stress technique for assessing myocardial perfusion in ischemic heart disease is myocardial contrast stress echocardiography (MCSE). Since perfusion abnormalities occur earlier than wall motion abnormalities, MCSE may be helpful in patients who cannot exercise, or those intolerants of high inotropic doses. It has higher sensitivity compared to single positron emission computer tomography (SPECT) (75.2% versus 49.1%) in the detection of CAD in a population with chest pain and high incidence of risk factors, particularly for the detection of microvascular disease [43, 44]. However, it has lower specificity compared to SPECT. Furthermore, high-dose dobutamine MCSE has shown similar diagnostic accuracy for diagnosing significant CAD, compared with CMR [45].

### Non-ischemic HF

In patients with HF symptoms and non-ischemic cardiomyopathy, various etiologies may be involved (cardiomyopathies, VHD, diastolic HF, cardiotoxicity, etc.). These conditions are relatively common and are associated with high mortality rate [59]. In early disease stages, when LV EF is still preserved, a reduction in the contractile reserve during SE identifies subclinical myocardial dysfunction, thus might help clarifying the diagnosis; HFpEF, VHD, or unexplained dyspnea [37]. SE should also assist in detecting early chemotherapy-induced cardiotoxicity [24], hypertensive as well as diabetic cardiomyopathy and thalassemia [37, 60–64].

On the other hand, in advanced HF residual myocardial contractile reserve assessed by SE can differentiate ischemic from non-ischemic cardiomyopathies, for prognostic stratification, and to guide clinical decision making [65]. Independent of LV EF, the absence of contractile reserve is often associated with limited CFR [66, 67], a marker of latent LV systolic dysfunction and sub-clinical cardiomyopathy. Moreover, the presence of inotropic contractile reserve has been associated with less need for cardiac transplantation [68, 69], being inversely correlated with the extent of interstitial fibrosis and scarred myocardium [70] and with better survival rate, fewer hospitalizations for HF [71], and increase in LV EF during follow-up [72]. A reduction or the absence of CFR and contractile reserve during dipyridamole SE in patients with non-ischemic cardiomyopathy proved to be a marker of poor prognosis [73, 74].

The addition of lung ultrasound to SE has provided useful in demonstrating HF. B-lines (or lung comets) are vertical, hyperechoic images that start from the pleural line and extend to the bottom of the display, without fading but move synchronously with respiration. In patients with HF, the presence and the number of B-lines has been shown to correlate with the presence of pulmonary interstitial edema and raised LV filling pressure [43, 75, 76]. During exercise SE, the appearance of B-lines might be used to demonstrate that exertional dyspnea is consequent to pulmonary congestion as a sign of HF [77].

The evaluation of B-lines has been proposed in the ABCDE-SE protocol introduced in the SE2020 study [78, 79]. This is a five-step protocol based on the shift in the pathophysiological model from stenosis vulnerability to patient vulnerability which emerged in the last decade, focusing not only on angina during the evaluation of SE. The currently ongoing SE2030 study is articulated in 12 different projects, in order to provide further evidence to finally recommend SE as the optimal and versatile imaging modality for functional testing [80].

#### Diastolic stress echocardiography

SE has been affirmed as a tool for detecting impaired LV diastolic function reserve and the subsequent increase in LV filling pressures [81–83] in patients with unexplained dyspnea or suspected subclinical diastolic dysfunction.

Its application is of additive value in patients with suspect HFpEF, in whom SE is currently recommended in the HFA-PEFF algorithm [84] as a component of the diagnostic workflow in cases of uncertainty (intermediate risk) after clinical assessments and standard diagnostic tests to confirm the diagnosis of HFpEF using a supine bicycle [84]. Post-exercise assessment should be performed during recovery, especially in patients with a rapid increase in HR at low level exercise. Moreover, a simple non-exercise modality for preload augmentation is passive leg raise, which may identify patients with raised stress-induced LV filling pressure causing lower exercise capacity [85] (Fig. S1).

The most investigated parameter in diastolic SE in previous studies is E/eʹ ratio as an index of filling pressures [86, 87]. To overcome the limit of high HR with E/A wave fusion, E/e’ ratio during low-level exercise (20W) proved to have higher feasibility and accuracy in predicting PCWP during peak exercise, hence supporting its use as an alternative to the peak exercise value in ruling out HFpEF in patients with dyspnea [88].

Another key marker for assessing LV diastolic function during SE is sPAP [8, 82, 89]. Exercise septal E/ e^′^ > 13, lower amplitude of changes in diastolic longitudinal velocities, and induced pulmonary hypertension (sPAP ≥ 50 mmHg) are markers of adverse outcomes in patients with HF [89–93].

However, E/eʹ may be misleading in the presence of mitral annular calcification, moderate/severe MR, constrictive pericarditis, mitral valve replacement/repair, left bundle branch block, or significant AR [91], and sPAP may be difficult to evaluate in the absence of TR or may be underestimated in the presence of a severe TR or impairment of RV function [89]. Therefore, new parameters have been evaluated, such as the ratio of early diastolic velocity of mitral inflow to the flow propagation velocity (E/Vp), marker of LV filling pressure, diastolic functional reserve index (DFRI), based on changes in e' velocity on exercise, isovolumic relaxation time (IVRT), LV diastolic strain rate and diastolic dyssynchrony. However, to date, these markers need to be validated [89]. For these reasons, currently, E/eʹ and sPAP are still the most used echocardiographic markers of LV filling pressure.

Another parameter which may be used to estimate DD is derived from the ratio between CO and invasive mean PCWP, a surrogate marker of left atrial pressure, both measured at baseline and during stress (Table 2), a stress marker of diastolic function compared to right heart catheterization [82, 85, 94–97]. However, the accuracy of the relationship between LV filling pressure and CO has been evaluated only in small and cross-sectional studies, and its clinical implications is limited [85, 94–98].

**Table 2:** Main parameters used for diastolic stress echo and their response to stress. ΔCO/ΔPCWPe, ratio of the variation in cardiac output to the variation in estimated pulmonary capillary wedge pressure derived from Nagueh’s formula^*^; A wave, late transmitral diastolic velocity; E wave, early transmitral diastolic velocity; e’, early TDI velocity of the mitral annulus; LV, left ventricle; SPAP, systolic pulmonary artery pressure; ^*^Nagueh’s formula: PCWP = 1.9 + 1.24 (E/eʹ)

DIASTOLIC STRESS ECHO			
**Parameter**	Response to stress	Cut-off	References
**e’**	= / ↑ Reduced suction reserve, increased diastolic stiffness	< 7 cm/s (septal); < 10 cm/s (lateral)	[84, 101–105]
**E wave**	↑↑ Increased diastolic stiffness, elevation of minimum LV diastolic pressure	-	[88, 92]
**E/e’**	↑↑ High LV end-diastolic pressure; PCWP	> 13	[87, 91–95]
**SPAP**	↑↑ High LV end-diastolic pressure, PH	> 35 mmHg at rest, > 43 mmHg at exercise	[85, 91, 92]
**ΔCO/ΔPCWPe**	↓ CO increases at the expense of an elevated PCWP	Grades of diastolic disfunction: - A (at risk): 0.8–1.0 - B (mild): 0.4–0.6 - C (severe): < 0.2	[84, 87, 96–100]
**Passive leg-raise**	↓E/A	< 1	[87]
	↑ E/e’	> 15	

### Valvular heart disease

The role of SE for the evaluation of VHD has been fully addressed in the latest guidelines of international societies [7, 103]. In these patients, symptoms may develop slowly, hence many patients may refer to be asymptomatic. For this reason, guidelines emphasize the use of exercise testing to assess objective evidence of symptoms and exercise capacity [7, 103]. Moreover, SE could be of value when there is discordance among baseline measurements and the symptoms [103]. In patients with prosthetic valves, disproportionate increase in mean transvalvular gradient during exercise (i.e. > 20 mmHg for aortic prostheses or > 12 mmHg for mitral prostheses) suggests severe prosthesis stenosis or significant patient-prosthesis mismatch [103]. Overall, SE is regarded a second line examination in VHD (usually after transthoracic and, sometimes, transesophageal echocardiography), to further assess etiology, severity, and prognosis [7]. In VHD, physical exercise is the test of choice since it provides the most physiological assessment, and a semi-supine bike is recommended [7, 104].

#### Mitral valve disease

SE may be useful to establish the etiology of MR (class of recommendation 1 for 2020 American guidelines) and to assess myocardial viability [7] in chronic secondary MR. It may also add prognostic information, as an increase in the effective regurgitant orifice area ≥ 13 mm^2^ during exercise is associated with higher risk of CV events in medically treated patients [105]; then, an increase in sPAP [105] and the development of exercise-induced B-lines [106] (signs of pulmonary hypertension and systemic hemodynamic congestion, respectively) are independently associated with higher rates of cardiac events in patients with MR. Marked changes in MR severity (at least 1 grade) [4] are associated with an increase in sPAP and reduced symptom-free survival [107].

Interestingly, Kusunose et al. have shown that in asymptomatic primary MR, RV function worsening during SE, shown as a tricuspid annular plane systolic excursion < 19 mm, might stratify prognosis independently of the onset of pulmonary hypertension [108].

In rheumatic mitral stenosis, SE is recommended to evaluate symptoms, exercise capacity, variation of mean mitral gradient and of sPAP [7]. An increase in mean transmitral gradient ≥ 15 mmHg or a sPAP ≥ 60 mmHg during exercise/stress is considered abnormal [103].

#### Aortic valve disease

In asymptomatic patients with severe AS, SE is recommended by international guidelines as it provides diagnostic and prognostic information beyond aortic valve area and transvalvular pressure and gradients at rest. In patients with suspected low-flow, low-gradient severe AS with reduced LV EF, low-dose dobutamine SE is recommended by ESC VHD guidelines (class I) to distinguish between true severe and pseudo-severe AS (increase in valve area to > 1.0 cm^2^) and identify patients with no flow (or contractile) reserve [7]. In patients with no flow reserve (increase in indexed SV ≤ 20%) “indeterminate AS severity” is diagnosed if aortic valve area and transvalvular gradient do not change. The absence of contractile reserve occurs in up to 30% of AS patients and is a predictor of high mortality in the perioperative period after surgical aortic valve replacement. [7] However, this pattern does not correlate with post-operative late survival, hence should not contraindicate surgical or percutaneous procedures as they improve long-term prognosis and LV function [7]. A fall in systolic BP ≥ 20 mmHg during SE and decreased exercise tolerance are indications of intervention (class IIa and I for 2020 American and 2021 ESC guidelines respectively) in patients with severe AS asymptomatic at rest.

Importantly, SE is not indicated and can be harmful (contraindicated, class III) in symptomatic patients with severe AS [7].

In patients with AR, neither exercise nor dobutamine SE is indicated to grade valvular disease severity, since the stress-induced increase in HR shortens diastole, therefore limiting the quantification of AR severity. Exercise echocardiography may help assessing the symptomatic status of the patients with severe AR, with a dramatic change in prognosis [9] and to evaluate LV contractile reserve [109]. In these patients, the timing of intervention could be anticipated in the presence of a lack of LV contractile reserve (defined as an increase in EF < 5%) [4].

#### Tricuspid regurgitation

SE could be used to assess exercise capacity in patients with severe tricuspid regurgitation (TR) irrespective of symptoms. It may aid the assessment of stress-induced worsening valve dysfunction, sPAP increase and myocardial ischemia. Furthermore, RV functional reserve may be assessed by measuring tricuspid annular plane systolic excursion (TAPSE), RV tissue doppler imaging S’, and RV fractional responses during stress [66]. RV longitudinal strain by speckle tracking echocardiography may also be measured, even though limited by high HR [110]. A decrease or lack of augmentation of these indices highlights an impairment in RV functional reserve and a worse outcome in patients with concomitant left-sided valve disease [111].

### Advanced heart failure—The ADONHERS protocol

Another potential application of SE in HF patients is in potential heart donors for patients with advanced HF, and thus is used as a mean to overcome the challenge of heart donor shortage in heart transplantation [112]. The ADONHERS protocol was developed in Italy to obtain a possible extension of eligible donors, despite age and non-significant comorbidities, applying an accurate screening by SE [6]. After excluding a severe LV hypertrophy or VHD and resting global or RWMT abnormalities, the brain-dead potential donor, aged > 55 (and less than 65) years or < 55 years with known multiple CV risk factors, undergoes dipyridamole SE. In the presence of preserved LV contractile function, assessed by systolic BP/LV end-systolic volume stress/rest ratio (Sagawa index) and normal global and regional function after stress, the potential donor can be considered eligible [113, 114]. Speckle-tracking echocardiography may be used as a valuable additional tool to overcome operator-dependency [115].

## Stress echo – new perspectives

### Contrast-enhanced stress echocardiography

**Fig. 2:**
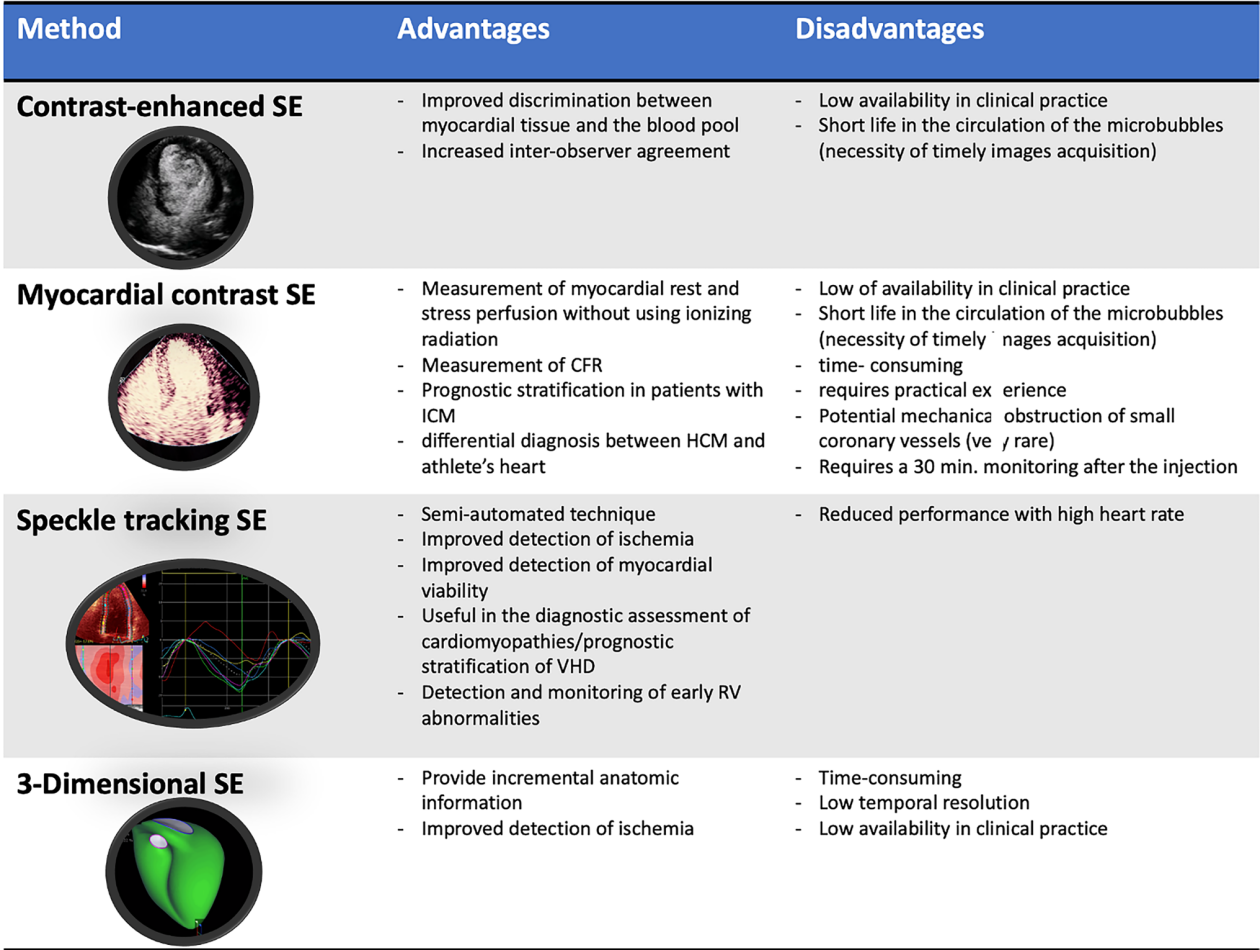
New perspectives in stress echocardiography. CFR, coronary flow reserve; HCM, hypertrophic cardiomyopathy; ICM, ischemic cardiomyopathy; RV, right ventricle; SE, stress echocardiography; VHD, valvular heart disease

Endocardial border definition may deteriorate at stress for translational heart movement produced by tachycardia or rapid chest wall movement (during exercise). To overcome these limitations, during SE, the intravenous injection of contrast agents, mainly microbubbles that contain air or high molecular weight gas may be used (Fig. 2). These help to identify RWMT abnormalities that could be missed, particularly in obese patients (sensitivity 91%, vs 80% of normal SE) [46]. Moreover, the use of contrast enhanced SE increases the inter-observer agreement (79% vs 69% in normal SE) [116] and improves the concordance of the interpretation of novice operators to the expert readers [117].

### Myocardial contrast stress echocardiography

MCSE can measure rest and stress perfusion of the myocardium without using ionizing radiation. It utilizes inert gas-filled microbubbles that entirely remain within the vascular space and have a rheology in the vessels like that of the red blood cells [118, 119]. This method provides an estimation of the two components of capillary perfusion: flow velocity and blood volume fraction, which product is proportional to CFR [120].

MCSE has shown promising results in the detection of ischemia, but it could also aid prognostic stratification of patients with HF and ischemic cardiomyopathy. Compared to clinical, LV functional parameters and SPECT, MCSE resulted as the only independent predictor of improvement of LV function in hibernating myocardium [121]. Vasodilator MCSE is capable of distinguishing physiological from pathological LV hypertrophy, since CFR is significantly reduced in patients with hypertrophic cardiomyopathy compared to athletes [122]. Finally, in patients with Tako-Tsubo cardiomyopathy, vasodilator MCE reveals a reversible perfusion defect, while the perfusion defect is persistent in patients with ST elevation myocardial infarction [123].

### Strain and strain rate stress imaging

The study of myocardial deformation during SE may also be of added value. Speckle tracking analysis is currently the most used and has been shown to overcome many tissue-Doppler limitations, above all angle-dependence and, being a semi-automated technique, poor inter-operator agreement [124–127]. LV global longitudinal strain (GLS) is most reproducible in all stages of dobutamine SE [128] and offers important additive information in ischemic HF, such as for the detection of ischemia, since the longitudinal subendocardial fibres are the first ones to be affected in the ischemic cascade, for the diagnosis of microvascular angina [124, 129]; for the assessment of viability in patients with severe LV systolic impairment; and for the differentiation between stunned and hibernating myocardium [130–132]. Furthermore, stress GLS during SE may be used for prognostic assessment of low-flow low-gradient AS, as it performs better than myocardial contractile reserve [133].

LV GLS can also be used in diagnostic assessment of cardiomyopathies, as it only slightly increases during stress in hypertension, and demonstrates significantly blunted augmentation in hypertrophic cardiomyopathy, with a similar absence of incremental increase in longitudinal diastolic function during exercise. Furthermore, time to peak strain measured during stress may be used for differential diagnosis in hypertrophic cardiomyopathy compared with hypertension [134]. Finally, STE could be an important tool in the detection and monitoring of early RV abnormalities. In arrhythmogenic RV dysplasia, RV strain values are reduced at rest and do not significantly increase during SE [135].

In dilated cardiomyopathy, the assessment of left atrial strain variation, as manifestation of functional reserve, has been proved to be an independent predictor of CV events. LA functional reserve could therefore act in people with HF as an additional prognostic tool together with LV contractile or diastolic reserve [136, 137].

However, speckle tracking during stress has some limitations (Fig. 2). Fast HR induced with stress, reduces image frame rate, 40–90 frames/sec, despite the overall accuracy it is described as only reasonable [128, 132]. Moreover, since in exercise SE post-ischemic RWMT anomalies persist even after recovery, speckle tracking has comparable sensitivity to that of conventional SE [138].

### Three-dimensional stress echocardiography

Three-dimensional (3D) echocardiography can be used with SE to provide incremental anatomic information, allowing direct visualization of RWMT and providing accurate measurements of LV ejection fraction and volumes. A correct acquisition of parasternal short axis view (PSAX) allows better appreciation of basal wall motion abnormalities, thus resulting in higher accuracy of 3D echocardiography compared to the multiplane mode, particularly for right coronary artery territory lesions [139]. Furthermore, by cropping the 3D volumetric dataset along the correct axes, the method avoids apical foreshortening, so becoming less likely to miss apical segments’ ischemia and LAD disease [140, 141]. Its main limitation is the low temporal resolution (Fig. 2). However, improvement of the technology has allowed the acquisition of the full volume in a single beat, with good image quality product (up to 40 volumes/sec) and elimination of artifacts related to body movement, increased respiration, or arrhythmias.

## Conclusions

SE plays an important role in the evaluation of HF patients, not only for accurate diagnosis but also for prognostic stratification and for planning therapeutic management strategies. The application of SE extends from the evaluation of ischemic HF, where it mainly detects myocardial ischemia and viability to providing significant therapeutic indications, to the study of non-ischemic HF. SE has also been recommended for diastolic HF and valvular pathologies, while further information is needed about the role of SE in patients with other cardiomyopathies, pulmonary hypertension, and on its impact on improving patient’s outcome (Central Illustration as Supplementary Material). Beyond being a bed-side, low-cost and highly available investigation, the high sensitivity and specificity of SE makes it an invaluable tool in the hands of the clinician. The use of SE with speckle tracking, contrast enhanced SE, MCSE and multidimensional imaging may overcome its limitations and further enhance its feasibility and additive value, thus paving the way for a further increase in its use in clinical practice, with significant benefit for HF patients.

### Supplementary Information

Below is the link to the electronic supplementary material.

Supplementary file1 (TIFF 8100 KB)

Supplementary file2 (TIFF 6548 KB)

## Data Availability

Not applicable.
