# Supplementary figures and images for: Stress echocardiography in heart failure patients: additive value and caveats
Source: Heart Fail Rev. 2024 Jul 26;29(5):1117–33. doi: 10.1007/s10741-024-10423-9 (PMC11306652; doi:10.1007/s10741-024-10423-9)

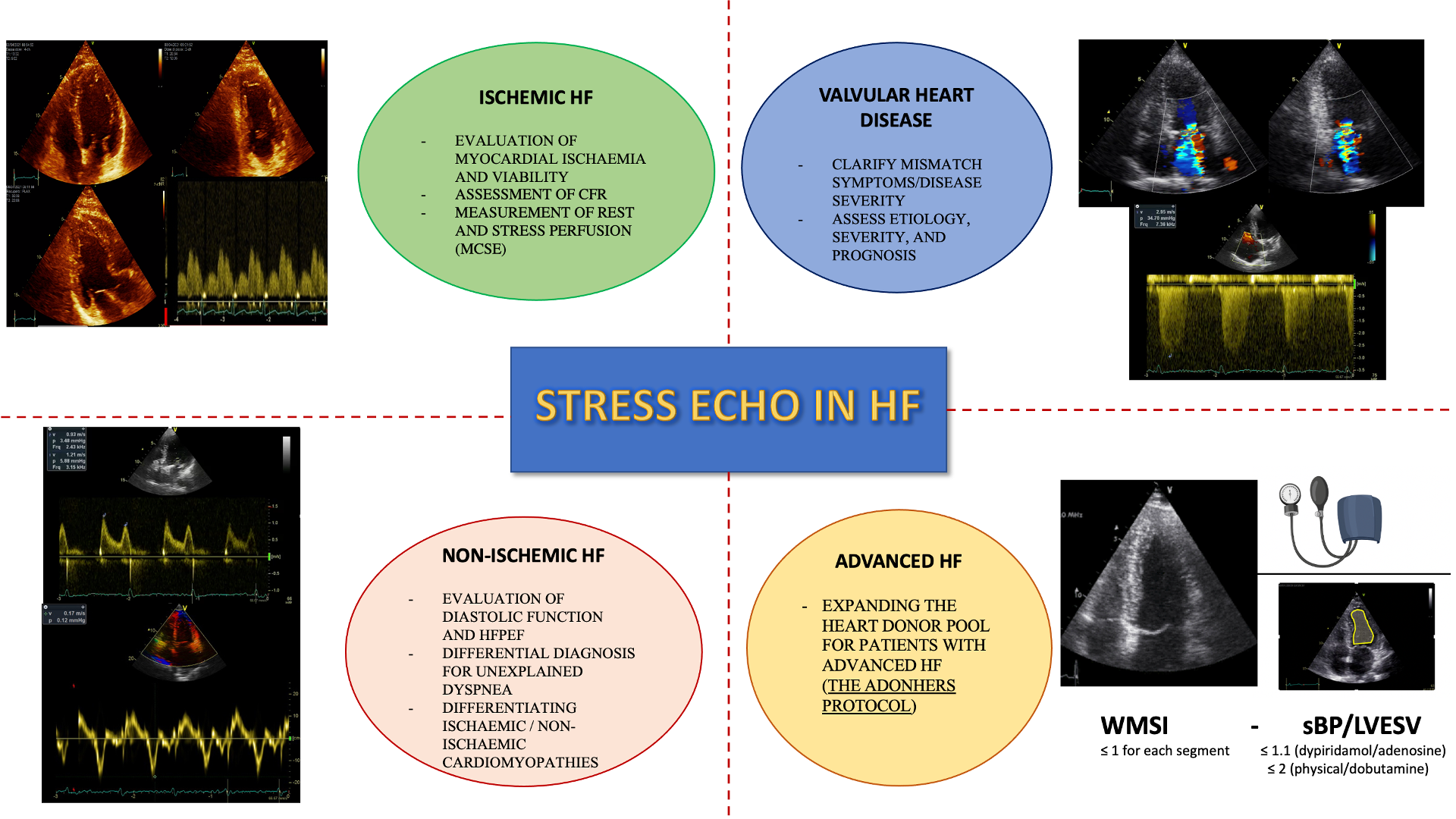

Supplement: Supplementary file 1 — Supplementary file1 (TIFF 8100 KB) [file 10741_2024_10423_MOESM1_ESM.tiff]

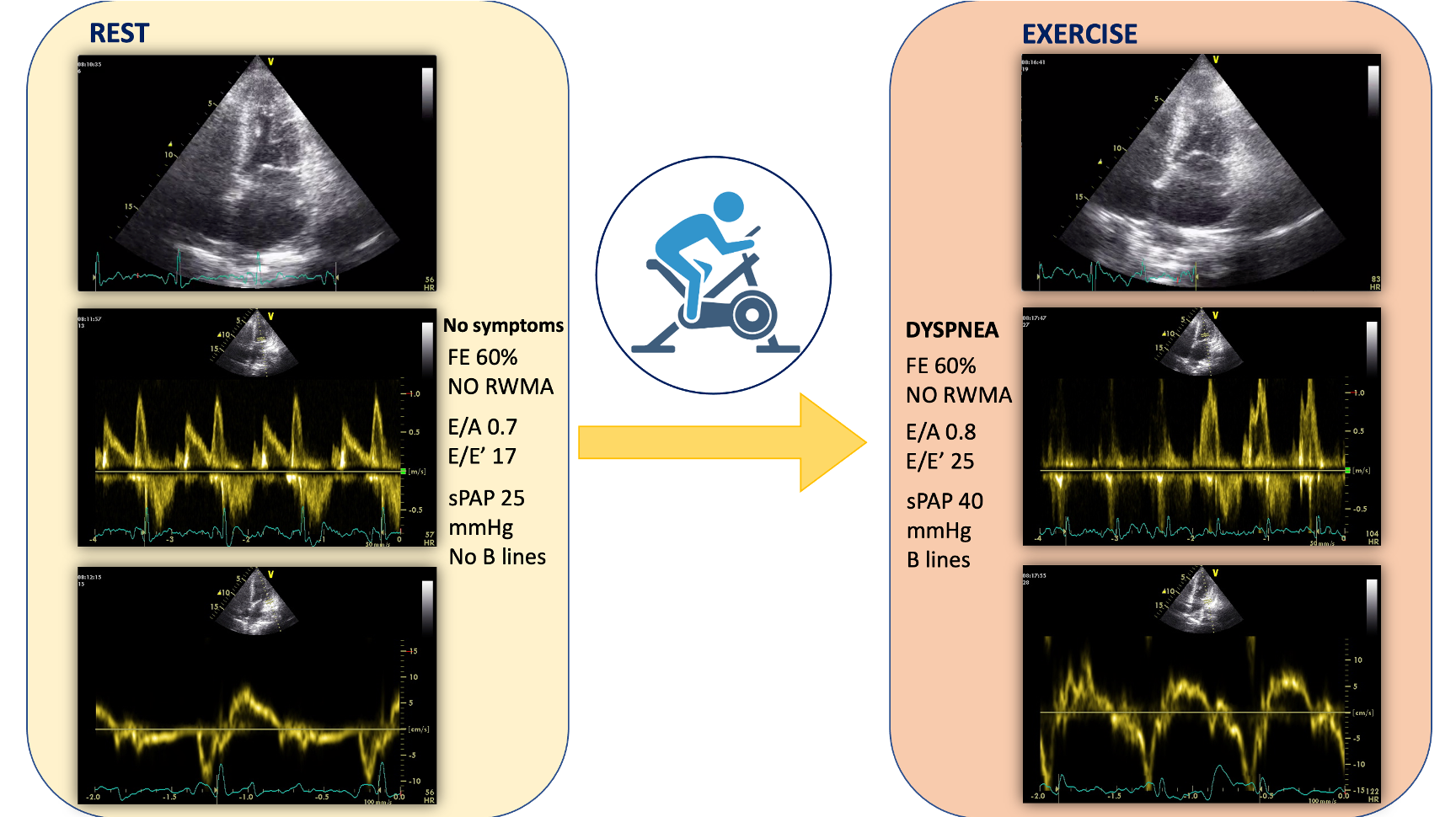

Supplement: Supplementary file 2 — Supplementary file2 (TIFF 6548 KB) [file 10741_2024_10423_MOESM2_ESM.tiff]
